# Primary health information standard system based on semantic interoperability

**DOI:** 10.1186/s12911-018-0696-5

**Published:** 2018-12-07

**Authors:** Xia Zhao, Xiaohua Li, Wei Yang, Qianjin Feng, Yi Zhou, Qiong Wang

**Affiliations:** 1 Guangdong Provincial Key Laboratory of Medical Image Processing, School of Biomedical Engineering, Sothern Medical University, Guangzhou, China; 2 General Hospital of Guangzhou Military Command of PLA, Guangzhou, China; 3 Sun Yat-sen University, Guangzhou, China; 4 The Third Affiliated Hospital of Guangzhou Medical University, Guangzhou, China

**Keywords:** Primary health information system, Primary health information standard system, Semantic interoperability, Date element, Dataset

## Abstract

**Background:**

To realize semantic interoperability for Primary Health Information System (PHIS), this study analyzes and applies existing health information data standards in China. This research aims to establish a Primary Health Information Standard System (PHISS), and achieve the semantic level interoperability and application of primary health information.

**Methods:**

First, the PHISS in accordance with the structural standards of national information standards system in China was constructed. Second, application of semantic interoperability level with reference to the interoperability model was standardized. Thirdly, referring to the data element model, PHIS data element dictionary with good interoperability is developed by standardizing data element attributes of identifiers, names, definitions and permissible value. Fourthly, based on PHIS data element dictionary, PHIS dataset is developed following the relevant rules for health information datasets.

**Results:**

PHISS is composed of basic class standards, data class standards, technical class standards, security and privacy class standards, management class standards. In this study, we reorganized the data class standards that meet the requirements of PHIS, also develops and adds PHIS data element, PHIS data element dictionary and PHIS dataset. PHIS data element dictionary includes 16 parts and PHIS dataset includes 22 parts, which satisfies the data standardization requirements of PHIS.

**Conclusions:**

The establishment of the PHISS can meet the needs for the interconnection of the residents’ basic health service information and realize the semantic level interoperability of various information services. The key steps of this method are based on semantic interoperability. Relevant data elements and datasets with semantic interoperability are selected. Moreover, an information standard system is constructed, and the information standardization requirements of the PHIS are met.

## Background

The primary health information system is an application system that aims to meet the needs of urban and rural residents for national basic health services. It can facilitate health record management, basic public health services, basic medical services, health information services, institutional operations for residents, and primary health supervision and management. Given its high requirements for information interoperability, the operation of the PHIS necessitates sharing and business collaboration with regional health information systems, medical institution information systems, medical insurance information systems, drug supply information platforms, health supervision and management information systems, as well as the data sharing and operation coordination of regional the PHIS. According to the “WS/T 517-2016 Fundamental Function Specification for Primary Health Information System”, PHIS standardization criteria include operational specification requirements, data standard requirements, technical specification requirements, and information security requirements [1]. To meet these standardization requirements will depend on the compliance and application of a series of related health information standards and regulations in China. The implementation of the primary health information standard system (PHISS) can meet the needs for the interconnection of residents’ basic health service information and realize the semantic level interoperability of various information services, supervision, and management, including public health record management, basic public health services, and health information services [2].

The information standard system is a scientific organism composed of a series of inter-connected standards within its scope. A standard system has purpose and coordination. Hence, each standard system revolves around a specific standardization requirement, which coordinates all standards within the system to be consistent and interconnected. The implementation of the PHISS can systematically categorize and apply complex health information standards and correctly select and utilize appropriate health information standards to meet the standardization requirements of the PHIS. In recent years, China’s National Health and Family Planning Commission issued more than 200 health information standards [3]. Therefore, choosing the standard specifications for the PHIS from the existing standard specifications and applying them effectively are key parts in constructing the PHISS.

Interoperability is an effective method to realize the interconnection among information systems, information platforms, and heterogeneous systems [4]. Interoperability in the field of health information provides an effective solution for the interconnection of such information. The basis of interoperability is standardization. The degree of standardization from primary technical interoperability to the higher level of semantic interoperability decides the levels of business collaboration and information sharing [5].

A dataset is a collection of data elements pertaining to a certain subject [6]. In the health domain in China, a dataset is usually based on the “WS 363-2011 Health Data Element Dictionary” (WS363–2011) [7]. Relevant data elements are extracted from the WS363–2011 according to the related business requirements and combined into a dataset.

Taking semantic interoperability as the goal, this study analyzes and applies the data standards of existing health information data in China, discusses the construction of the PHISS, develops the PHIS element dictionary and PHIS basic dataset.

## Methods

### Information standard system

**Fig. 1:**
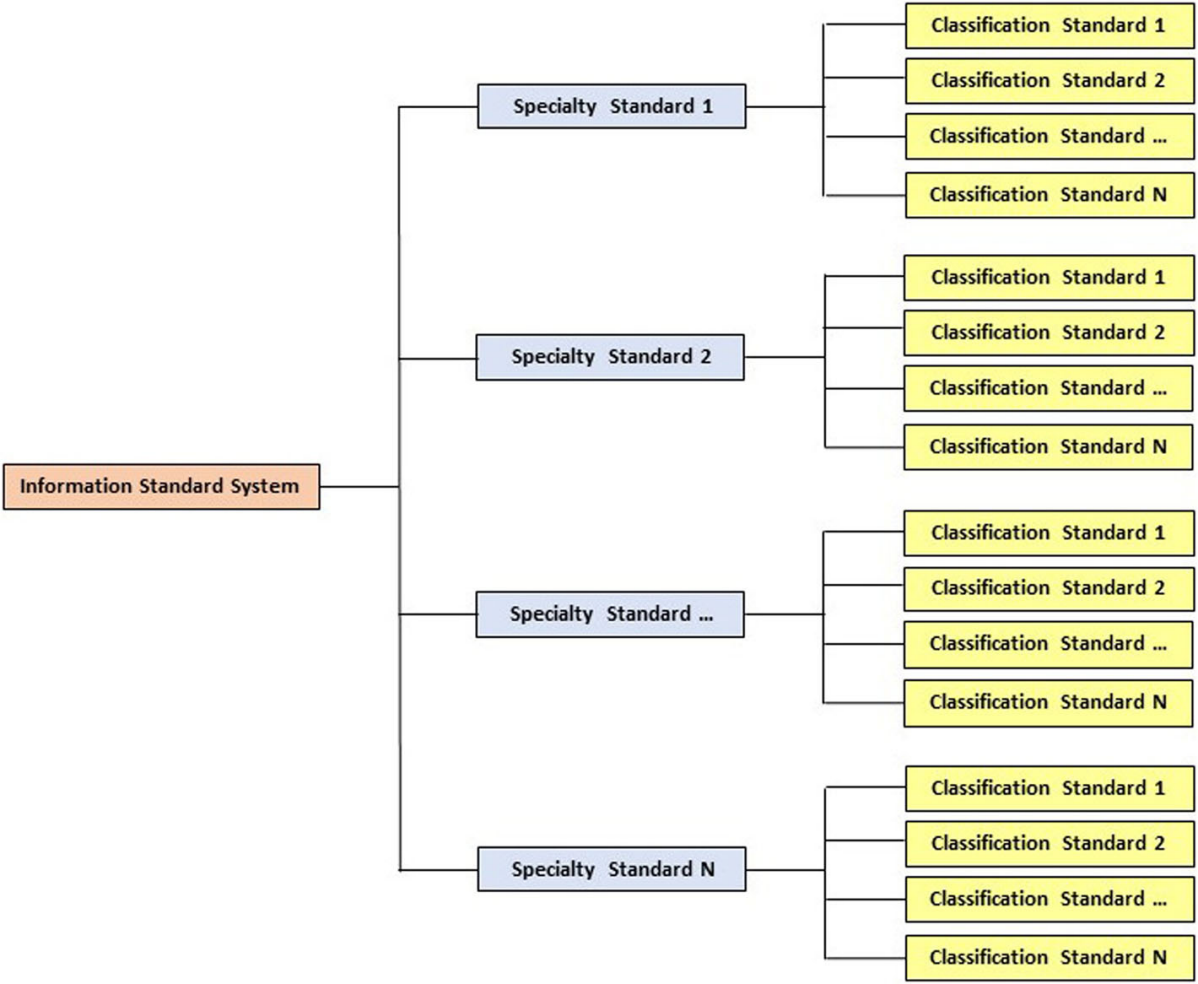
Standard architecture diagram

In accordance with the “GB/T 13017-2008 Guidelines for Preparing Diagrams of Enterprise Standard System”, the standard system consists of a standard architecture diagram and a standard system detail list [8]. The standard architecture is divided into three forms: the hierarchical, functional, and sequence structures. The first two are applicable to comprehensive or global management, and the third one is applicable to special or local management. The project in this study adopts a hierarchical structure-standard architecture, as shown in Fig. 1.

The standard system detail list consists of the following nine items: serial number, standard code and number, appropriate grade, standard name, implementation date, the international or foreign standard code implemented or adopted, corresponding relationship, review opinions, the replaced or invalidated standards code, and remarks. The original list can also be reduced to a simple one. Compared with the former, the simple list retains the following four items: serial number, standard code and number, standard name, and notes (Table 1).

**Table 1:** Standard specification (simple list)

SERIAL NUMBER	CODE OF STANDARD	NAME OF STANDARD	REMARK

### Semantic interoperability

**Fig. 2:**
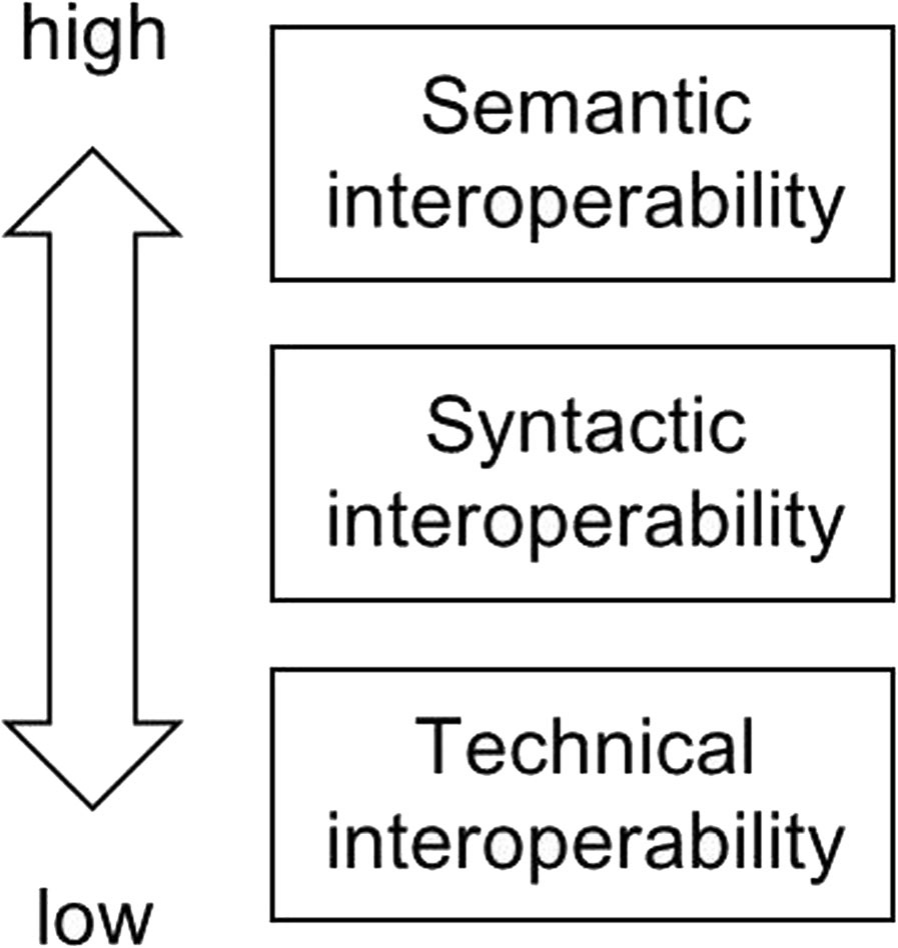
Interoperability levels

According to the definition from the IEEE Standard Computer Glossary, interoperability is the ability of two or more systems or components to exchange information and to use the information that has been exchanged [9]. This description refers to interoperability including two aspects of capabilities: the ability to exchange information (syntactic interoperability) and the ability to interpret exchanged information (semantic interoperability). Fig. 2 shows the hierarchical levels of interoperability.

The technical level refers to the creation of physical connections such that data can be exchanged between systems. Here, data exchange means that digital signals can be transmitted between systems. At the technical level, the system for exchanging data does not know or care about the content exchanged, does not require domain-specific expertise, and exchanges messages and files regardless of content. The syntactic level representation supports the same protocol and format, and data can be exchanged in a standardized format. The semantic level realizes that the meaning of the data is defined by a common reference model, and the data and its context can interact to correctly understand the information received from the other party [10].

The health information standards related to semantic interoperability mainly include the following: medical terminology standards, metadata and data elements, information models, and datasets etc. These standards play an important role in achieving the semantic interoperability of information exchange between systems [11]. In recent years, China issued a series of health information standards that provide strong support for the establishment of the PHISS. However, given the different standards and application environments, not all standards are applicable to the PHISS. Therefore, In establishing PHISS, in addition to referencing and citing existing standards that conform to PHIS interoperability, PHIS data element dictionary and PHIS dataset with good interoperability need to be developed and supplemented [12].

### PHIS data element

When selecting data elements, we need to consider whether the generation of data elements is based on a particular business or based on a business domain model [13]. Data element formation must be tracked, and its metadata or meta model be analyzed to understand the interoperability of data elements or data element dictionaries [14]. If the data element is based on a specific business, then it may cause ambiguity when used for integrated services or cross-over with other standards [15]. PHIS data element follows the data element model, defined by “WS/T303-2009 Rules for Data Element Standardization of Health Information” [16].

PHIS data elements consist of following two parts:

- The national health data element standard that has been published, such as the WS363–2011. It includes 1400 basic data elements of health information. These data elements are divided into 16 parts, including clinical examination, medical diagnosis, medicines and equipment, and health management, etc.
- Lacking data elements in national standards are supplemented, according to the information standardization requirements of PHIS. In keeping with the WS363–2011, these added data elements contain six main attributes:
- Data identifier, data element name, definition, data type, format, and permissible values. The data identifiers, names, definitions, and permissible values are given priority. These four types of attributes have strong semantic interoperability.

#### Data identifier

**Fig. 3:**
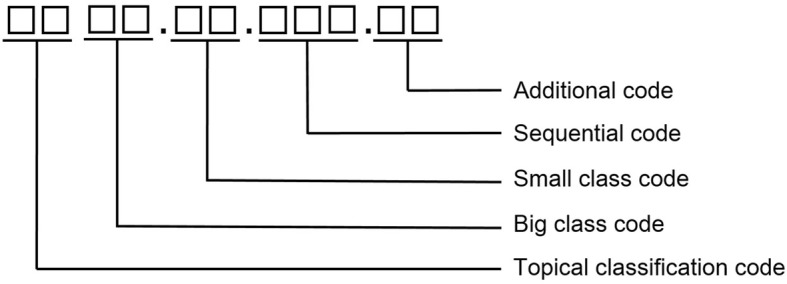
Structure of the data identifier

Referring to the identifier coding rules of WS363–2011, the PHISS data element directory has identical identifiers with the national standard specifications. The structure of the data identifier in the PHISS data element directory is illustrated, as shown in the Fig. 3.

#### Data element name and definition

Data element names and definitions need to refer to the relevant medical terminology standards, using the consensus data names, definition and concepts in the health domain, striving to standardize and uniqueness, to minimize the possible ambiguity when using data elements. Standardized terminology standards play an important role in the contextual understanding of the implementation of semantic interoperability [17]. Therefore, performing semantic analysis on the names and definition attributes of the data elements is necessary to check whether the CMV is followed, such as the SNOMED CT. Table 2 lists the terminology standards referred to in the supplementary data element.

**Table 2:** The supplementary data element reference terminology standards list

Code of standard	Name of standard
GB/T 16751–1997	Clinic Terminology of Traditional Chinese Medical Diagnosis and Treatment
SNOMED CT	Systematized Nomenclature of Medicine - Clinical Terms
GB/T 14396–2016	Classification and Codes of Diseases
ICD-10	International Classification of Diseases
WS/T 102–1998	Classification and Codes of Clinical Laboratory Tests Item
LOINC	Logical Observation Identifiers Names and Codes

#### Permissible value

The permissible values of data elements can judge the state and result of a data and also play an important role in semantic interoperability. Table 3 lists the standards for reference when adding data element permissible values. Because the relevant criteria of permissible values are not enough, this study also refers to the relevant clinical practice standards and guidelines in China.

**Table 3:** the supplementary data element permissible values reference standard list

Code of standard	Name of standard
WS 364–2011	Classification and Coding for Value Domain of Health Data Element
WS 446–2014	Codes for Common Laboratory Tests in Health Records
Release in progress	Classification and Coding for Value Domain of Health Data Element (Traditional Chinese medicine, TCM)

### PHIS basic dataset

On the basis of PHIS data element directory, PHIS basic dataset is compiled. In the process of compiling the PHIS basic dataset, besides according to the PHIS data element catalogue, a series of national health basic dataset standards related to PHIS business are also consulted, as shown in Table 4. WS 370–2012, WS/T 305–2009 and WS/T 306–2009 are used to standardize the compilation of basic dataset of PHIS, and other Basic Datasets are used for reference when compiling datasets of PHIS-related business parts respectively.

**Table 4:** Basic dataset reference standards list

Code of standard	Name of standard
WS 370–2012	Rules for Health Information Dataset Classifying and Coding
WS/T 305–2009	Metadata Specification of Health Information Dataset
WS/T 306–2009	Rules for Health Information Dataset Classifying and Coding
WS 371–2012	Basic Dataset of Basic Information
WS 365–2011	Basic Dataset of Health Record for Residents
WS 445–2014	Basic Dataset of Electronic Record
WS 376–2013	Basic Dataset of Children’s Health
WS 377–2013	Basic Dataset of Women’s Health
WS 373–2012	Basic Dataset of Clinical Healthcare
WS 372–2012	Basic Dataset of Disease Management
WS 375–2012	Basic Dataset of Disease Control

In the semantic interoperability, data standards of information standard systems play a key role. Hence, this research focuses on the analysis and application of the data standards. Other types of standards for PHIS will be introduced in other papers of this study.

## Results

### PHISS architecture

**Fig. 4:**
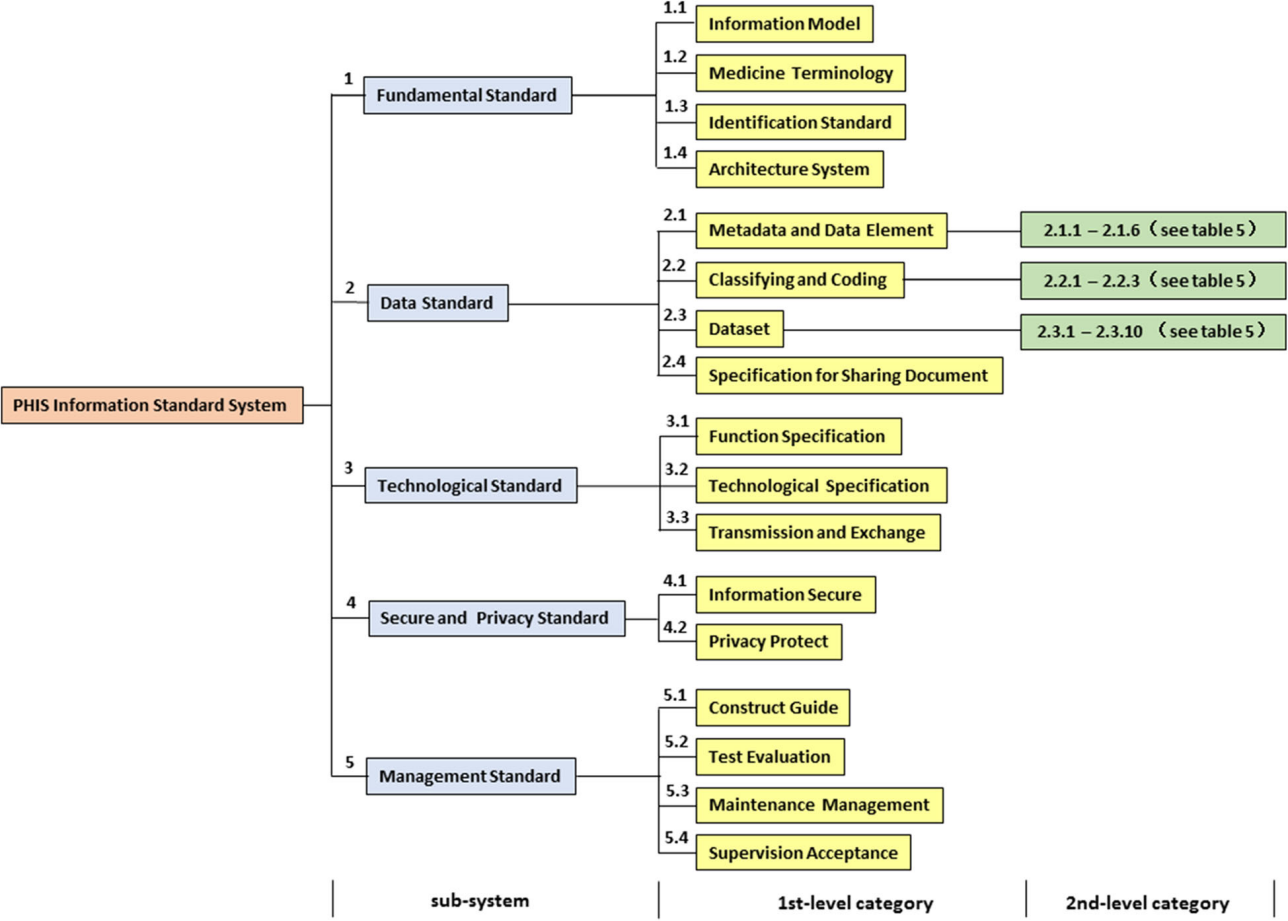
PHISS architecture diagram. From left to right, the figure shows the hierarchical expansion of standard system: standard classes (sub-system), standard classification (1st-level category) and standard document (2nd-level category). The numbers in the Fig. 4 represent the serial number of PHISS (see Table 5)

By referring to the basic framework of the health information standard system proposed by the Health Information Standardization Committee of the Health and Family Planning Commission in China [18], the PHISS architecture is designed in a hierarchical structure, as shown in Fig. 4.

The PHISS consists of five standard classes: fundamental standards, data standards, technological standards, security and privacy standards, and management standards [19]. Each class includes several secondary categories and can be extended to N category levels. The depth of the category level meets the requirements of standardization and the basic function of PHIS.

### PHIS standard list

The first step in the preparation of the standard list is to collect and collate relevant domestic and international health standards that meet the PHISS architecture category. But the existing health information standards may not fully meet the requirements of the PHISS, some standard specifications must be developed and supplemented, for example, Specification for Drafting of PHIS Dataset, PHIS Data Element Dictionary, PHIS Basic Dataset. These additional standards should also be included and noted in the list. Table 5 is the simple list of PHIS for data class standard.

**Table 5:** PHIS standard simple list (data class standard)

SERIAL NUMBER	CODE OF STANDARD	NAME OF STANDARD	REMARK
2.1.1	WS/T 305–2009	Metadata Specification of Health Information Dataset	
2.1.2	WS/T 306–2009	Rules for Health Information Dataset Classifying and Coding	
2.1.3	WS 370–2012	Specification for Drafting of Health Information Dataset	
2.1.4	Supplement	Specification for Drafting of PHIS Dataset	
2.1.5	WS363–2011	Health Data Element Dictionary	
2.1.6	Supplement	PHIS Data Element Dictionary	
2.2.1	WS 364–2011	Classification and Coding for Value Domain of Health Data Element	
2.2.2	WS 446–2014	Codes for Common Laboratory Tests in Health Records	
2.2.3	Release in progress	Classification and Coding for Value Domain of Health Data Element (TCM)	
2.3.1	WS 445–2014	Basic Dataset of Electronic Medical Record	
2.3.2	WS 365–2011	Basic Dataset of Health Record for Residents	
2.3.3	WS 371–2012	Basic Dataset of Basic Information	
2.3.4	WS 372–2012	Basic Dataset of Disease Management	
2.3.5	WS 373–2012	Basic Dataset of Clinical Healthcare	
2.3.6	WS 374–2012	Basic Dataset of Health Management	
2.3.7	WS 375–2016	Basic Dataset of Disease Control	
2.3.8	WS 376–2013	Basic Dataset of Children’s Health	
2.3.9	WS 377–2013	Basic Dataset of Women’s Health	
2.3.10	Supplement	Basic Dataset of PHIS	

The three-digit serial number given by x.x.x in the first column of Table 5 indicates the following: the subsystem number, the first-level category number, and the second-level category number. For instance, the serial number 2.3.2 denotes, from left to right, respectively, the data class standard (sub-system), the dataset (first-level category), and the basic dataset of the residents’ health records (second-level category). If a subordinate level category exists, then the sequence number is expanded to the right.

### PHIS data element dictionary

According to the basic principles and methods of data element standardization, the basic and universal data element standards are established and catalogued following the construction of PHIS database and the needs of data exchange, sharing, service and application of PHIS for data element. Table 6 lists the classification of PHIS data element dictionary.

**Table 6:** classification of PHIS data element dictionary list

Part number	Classification of data element
Part1	Identification
Part2	Demographics and social economics characteristics
Part3	Health history
Part4	Health risk factor
Part5	Chief complaint and symptom
Part6	Physical examination
Part7	Assistant examination
Part8	Laboratory examination
Part9	Diagnosis
Part10	Medical assessment
Part11	Medical plan and intervention
Part12	Healthcare expenditure
Part13	Healthcare organization
Part14	Health personnel
Part15	Drug, equipment and material
Part16	Health management

### PHIS basic datasets

Datasets are widely used in the standardization of health information in China. PHIS basic datasets are the basis of information exchange among different systems. They can also be used as a reference for information system developers in data structure and database design. Table 7 is the classification of PHIS basic dataset.

**Table 7:** Classification of PHIS basic dataset

Part number	Classification of dataset
Part1	Personal information
Part2	Medical institution information
Part3	Medical record summary
Part4	Outpatient and emergency medical record
Part5	Outpatient and emergency prescription
Part6	Examination and laboratory test record
Part7	Admission record
Part8	Nursing valuation and plan
Part9	Nursing operation record
Part10	General therapy and treatment record
Part11	Informing information
Part12	Inpatient order
Part13	Inpatient progress note
Part14	Delivery record of therapy and treatment
Part15	Home page of inpatient medical record
Part16	Home page of inpatient medical record summary of TCM
Part17	Discharged brief
Part18	Transfer record
Part19	children’s health
Part20	women’s health
Part21	Disease management
Part22	Disease control

## Discussion

The PHIS covers residents’ health records and patient electronic medical record management, basic public health services, basic medical services, health information services, institutional operation management, and grassroots health supervision. It involves numerous standards and a wide range of standardization requirements. The application of the PHISS provides an effective solution for the standardization needs of the PHIS. In establishing the PHISS, the following points must be considered:

### Hierarchy of the architecture

The design of the standard architecture should be in accordance with the standard system and refer to the overall design and basic functions of the PHIS according to the standardization requirements of the PHIS. The hierarchy of the standard system (category level) should cover all the standardization requirements of the PHIS and provide standardized support for the basic functions of the PHIS. The category level extends to the basic function point of the PHIS and can be gradually added in the implementation.

### Scope of the standard details

Presently, hundreds of types of information standards related to health in country, industry, and local can be collected in China in addition to foreign standards. However, the standard system is not a standard collection, but a careful classification and selection of standards closely related to the PHIS according to the actual needs of the PHISS. First, national standards (GB) and health industry standards (WS) that are directly related to the PHIS must be followed. Second, the health industry recommendation standards (WS/T) and local standards (DB) should also be complied with. Third, international recognized standards in health domain should be carefully referenced. Fourth, the relevant documents issued by the National Health and Family Planning Commission should be included in the standard system if they have guidance and normative effects on the PHISS. Finally, the primary election can entail a multiple selection, and then gradual screening can be conducted to form a clear, practical, and effective PHISS standard list.

### Semantic interoperability of data element attribute

In the preparation of the PHISS, the goal is to achieve semantic interoperability, that is, to strive for a system that understands the information received from other systems and uses and interprets the information unambiguously. An attribute refers to the characteristics of an entity or object. Health information data element attributes are generally divided into six categories: identification class, definition class, relationship class, identity class, presentation class, management class, and additional class. Among the six types of attributes, the most commonly used health data standards are the identifier and name of the identification class; the definition of the definition class; and the permissible value of the presentation class. These attributes are important for semantic interoperability. If ambiguities are found in the related attributes in different standards, then they must be solved by mapping [20]. For instance, if the data element identifier adopts different coding systems, then the method of establishing a mapping table can be used to implement semantic matching data conversion. But this method will reduce the efficiency of data transmission and exchange.

### Dataset application

Dataset is a set of data elements with a certain theme. Datasets are characterized by a specific healthcare business oriented to address specific business data standardization applications. The data elements of a dataset usually come from a data element directory, such as WS365–2011 for Residents, which comes from WS363–2011, and can be added if necessary. The development of PHIS basic dataset is based on the national health information basic dataset compilation specification. It refers to the basic datasets of various health care services related to PHIS services, and makes it meet the data standardization requirements of PHIS, and has good semantic interoperability with other healthcare information systems.

### Standard supplement

For a new health information application, existing information standards are unlikely to fully meet the requirements of the new application. In this case, developing and supplementing on the basis of existing relevant standards are necessary. Given that the PHISS involves the application of basic public health services, medical services, and Chinese medicine services in a PHIS system, the relevant data element, data element dictionary and datasets have been developed.

### Limitations of existing standard systems

In establishing the PHISS, the author believes that the standardization of health information should be strengthened in the following aspects. First, the quantity of standard need be increased. Although a series of related health information standards has been released in China in recent years, it is subdivided into each professional field, and the number of standards is insufficient. Second, the semantic interoperability between standards must be enhanced because the health industry involves many professional fields, the current coordination of information standards in each professional field is insufficient, and semantic interoperability is overlooked. For instance, data identification is inconsistent and non-standard, thereby creating barriers to cross-domain applications. Third, some standards lack metadata specifications, and analyzing the semantic interoperability of the standard from the level of data model and conceptual model is difficult. Fourth, the lack of research and application of terminology standards affects the improvement of semantic interoperability, and the use of internationally recognized terminology standards, such as SNOMED CT, should be promoted in China.

## Conclusions

It is an effective method to solve the information standardization requirement of PHIS by establishing PHISS. The key steps of this method are based on semantic interoperability. Normalizing the data element attribute, keeping the CMV and guiding by the information model are three conditions in the selection of relevant data elements and datasets with semantic interoperability from the current national, industry and local health information standards. By this way, the relevant data elements and datasets which have semantic interoperability are selected. The information standard system is constructed to meet the information transmission and sharing requirements of PHIS.

Unlike the usual modeling methods, our study is based on the health information standards promulgated in China. According to the business functions and interoperability requirements of the PHIS, data elements and dataset standards as essential factors are selected to constitute the PHISS. The method is compact and effective. The PHISS is also based on existing health information standards in China and it has good interoperability. Its shortcoming is the lack of information modeling guidance, the tendency to overlap or encounter cross-over issues, and the decrement on the data exchange and sharing at the semantic level.

## Abbreviations

CMV: Controlled Medical Vocabulary
PHIS: Primary Health Information System
PHISS: Primary Health Information Standard System
