# Correction to: Primary health information standard system based on semantic interoperability

**DOI:** 10.1186/s12911-019-0861-5

**Published:** 2019-07-25

**Authors:** Xia Zhao, Xiaohua Li, Wei Yang, Qianjin Feng, Yi Zhou, Qiong Wang

**Affiliations:** 1 Guangdong Provincial Key Laboratory of Medical Image Processing, School of Biomedical Engineering, Southern Medical University, Guangzhou, China; 2 General Hospital of Guangzhou Military Command of PLA, Guangzhou, China; 3 Sun Yat-sen University, Guangzhou, China; 4 The Third Affiliated Hospital of Guangzhou Medical University, Guangzhou, China

**Correction to: BMC Med Inform Decis Mak**

**https://doi.org/10.1186/s12911-018-0696-5**

After publication of this supplement article [1], it was brought to our attention that the first and correspondence authors’ affiliation information was incorrectly spelt. The original spelling was written as ‘Sothern’. However, the correct spelling should be ‘Southern’.

The correct affiliation of the first and correspondence authors is ‘Guangdong Provincial Key Laboratory of Medical Image Processing, School of Biomedical Engineering, Southern Medical University, Guangzhou, China’.
